# Improving cancer immunotherapy in prostate cancer by modulating T cell function through targeting the galectin-1

**DOI:** 10.3389/fimmu.2024.1372956

**Published:** 2024-06-17

**Authors:** Hsiao-Chi Wang, Roger Xia, Wen-Hsin Chang, Ssu-Wei Hsu, Chun-Te Wu, Ching-Hsien Chen, Tsung-Chieh Shih

**Affiliations:** ^1^ Department of Research and Development, Kibio Inc., Houston, TX, United States; ^2^ Department of Biomedical Data Science, Stanford University, Stanford, CA, United States; ^3^ Division of Nephrology, Critical Care and Sleep Medicine, Department of Internal Medicine, University of California, Davis, Davis, CA, United States; ^4^ Division of Pulmonary, Critical Care and Sleep Medicine, Department of Internal Medicine, University of California, Davis, Davis, CA, United States; ^5^ Comprehensive Cancer Center, University of California, Davis, Davis, CA, United States; ^6^ Department of Urology, Chang Gung Memorial Hospital, Linko, Taiwan; ^7^ Department of Translational Molecular Pathology, The University of Texas MD Anderson Cancer Center, Houston, TX, United States

**Keywords:** galectin-1, TME, immunotherapy, LLS30, prostate cancer

## Abstract

Our study aimed to elucidate the role of Galectin-1 (Gal-1) role in the immunosuppressive tumor microenvironment (TME) of prostate cancer (PCa). Our previous findings demonstrated a correlation between elevated Gal-1 expression and advanced PCa stages. In this study, we also observed that Gal-1 is expressed around the tumor stroma and its expression level is associated with PCa progression. We identified that Gal-1 could be secreted by PCa cells, and secreted Gal-1 has the potential to induce T cell apoptosis. Gal-1 knockdown or inhibition of Gal-1 function by LLS30 suppresses T cell apoptosis resulting in increased intratumoral T cell infiltration. Importantly, LLS30 treatment significantly improved the antitumor efficacy of anti-PD-1 *in vivo*. Mechanistically, LLS30 binds to the carbohydrate recognition domain (CRD) of Gal-1, disrupting its binding to CD45 leading to the suppression of T cell apoptosis. In addition, RNA-seq analysis revealed a novel mechanism of action for LLS30, linking its tumor-intrinsic oncogenic effects to anti-tumor immunity. These findings suggested that tumor-derived Gal-1 contributes to the immunosuppressive TME in PCa by inducing apoptosis in effector T cells. Targeting Gal-1 with LLS30 may offer a strategy to enhance anti-tumor immunity and improve immunotherapy.

## 1 Introduction

Prostate cancer (PCa) stands as the prevalent malignancy and ranks as the second-leading cause of cancer-related mortality in men. Initial treatment for prostate cancer involves androgen deprivation therapy (1). However, a significant challenge arises for patients facing metastatic castration-resistant prostate cancer (mCRPC). mCRPC poses a critical issue, marked by a poor prognosis, and a majority of patients typically undergo disease recurrence within a span of 16 to 18 months (2). Most conventional anticancer therapies and immunotherapy are designed to specifically target PCa cells. However, the immunosuppressive tumor microenvironment (TME) in PCa plays a crucial role in promoting resistance to these therapies (3). For example, T-cell exclusion is prominent in PCa, limiting direct engagement with cancer cells. Immune cells are confined to adjacent stroma and benign areas, often exhibiting immunosuppressive traits (4, 5). This microenvironment is driven by elevated levels of indoleamine 2, 3-dioxygenase (IDO), nitric oxide (NO), interleukin 10 (IL10), transforming growth factor-beta (TGF-β), arginase and adenosine (5–8). Therefore, the development of strategies that target the TME emerges as an attractive approach for the effective treatment of PCa.

The 135 kDa Galectin-1 (Gal-1) protein is encoded by the gene *LGALS1* at 22q13.1 (9). Gal-1 is a family of carbohydrate-binding lectins that bind to β-galactoside-containing glycoconjugates (10). Gal-1 is upregulated in cancers (11–15), located both extracellularly and intracellularly, and contributes to many cancer related events including cell proliferation (16), T cell apoptosis (17, 18), angiogenesis (19), and metastatic spread of cancer (20–22). Several Gal-1-binding proteins have been discovered, including H-Ras, integrins, laminins, fibronectin, vitronectin, osteopontin, neuropilin-1, CD45, CD146, and CD326 (23, 24). For example, extracellular Gal-1 recognizes terminal galactose residues β-1,4-linked to LacNAc, which is present in CD45 T cell receptors. Through the binding of LacNAc, Gal-1 can stimulate apoptosis of effector leukocytes (17). Extracellular Gal-1-binding usually occurs through protein-carbohydrate interactions which could be inhibited by lactose (25). In our previous study, we demonstrated that Gal-1 is upregulated in PCa patients and is highly expressed in CRPC cells, but not in androgen-sensitive cells (26). In addition, elevated levels of Gal-1 were observed in the extracellular matrix, and increased stromal Gal-1 expression is associated with poorer outcomes (27). These findings illustrate the importance of Gal-1 in PCa TME. However, whether Gal-1 secreted by PCa cells and its impact on T cell death remain unexplored.

Given that Gal-1 expression in PCa is associated with adverse clinical outcomes, the inhibition of Gal-1 should be considered as a potential treatment approach for CRPC. To target Gal-1, we developed a novel small molecule inhibitor of Gal-1 named LLS30 (26). LLS30 is a benzimidazole-based small molecule with the ability to inhibit the growth and metastasis of PCa xenografts in athymic mice (26, 28, 29). LLS30 functions as a Gal-1 allosteric inhibitor and leads to a decrease in the binding affinity of Gal-1 to its partners. Notably, LLS30 potentiates the antitumor effect of docetaxel and leads to a complete regression of CRPC tumors cells *in vivo* (26). Furthermore, LLS30 exhibits the capability to inhibit tumor invasion and metastasis *in vivo* (26).

In this study, our research results demonstrated the secretion of Gal-1 by PCa cells and its potential to induce T cell apoptosis. Furthermore, Gal-1 inhibitor LLS30 was found to significantly suppress the Gal-1 induced T cells apoptosis *in vitro* and *in vivo*. Importantly, LLS30 can significantly enhance the antitumor efficacy of anti-PD-1 *in vivo*. These findings illustrate a potential mechanism of immusuppressive TME in PCa and provide a strategy to improve the therapy outcomes for immunotherapy resistant PCa.

## 2 Materials and methods

### 2.1 Cell lines

The cell lines include human AR-positive 22RV1 and AR-negative PC3, as well as mouse AR-positive Myc-CaP PCa cells. These cells were cultured in RPMI1640 (22RV1 and PC3) or DMEM (Myc-CaP), supplemented with 10% fetal bovine serum and 1% penicillin/streptomycin, and maintained in a 5% CO_2_ atmosphere at 37°C. Routine monthly testing for Mycoplasma contamination was conducted.

### 2.2 Patients samples

Patient samples were obtained from US Biomax (PR803b, 66cases) and the University of California Davis Comprehensive Cancer Center (83cases) under the protocol titled ‘De-identification and Usage of Existing Pathology Specimens for Research Purposes by the UC Davis Pathology Biorepository,’ with the Institutional Review Board number 293828. Gleason grading was done on tissue core based on 2005 ISUP modified system (30): Gleason score<6 (low grade), = 7 (intermediate grade), and >8 (high grade). Human tissues covered 38 cases of benign hyperplasia (BHP), 32 cases of low grade, 24 cases of intermediate grade and 55 cases of high grade PCa.

### 2.3 Co-cultures of PCa cells with peripheral blood mononuclear cells

To initiate the experiment, 5 × 10^3^ 22RV1 cells were seeded in each well of a 48-well plate containing 100 μL of complete culture medium (RPMI1640 supplemented with 10% fetal bovine serum and 1% penicillin/streptomycin). The plates were then left to incubate overnight, allowing the cells to adhere, a process taking approximately 24 hours. By the following day, it was estimated that the number of cancer cells had doubled to 1 × 10^4^ cells per well, forming the basis for calculating Effector-to-Target (E:T) ratios. For co-culture experiments, PBMCs serving as effector cells, were mixed with 22Rv1 cells at a ratio of 3:1. PBMCs were cultured in complete medium and activated with IL-2 (5 ng/mL). After 24 hours of activation, PBMCs were preincubated with anti-PD-1 (Nivolumab, selleckchem) at concentrations of 10 μg/ml, 20 μg/ml, and 40 μg/ml for 30 minutes. Subsequently, they were added to the cancer cells in the presence or absence of 1 μM or 2 μM LLS30 for an additional 24 hours. The culture with PBMCs and 22RV1 cells without nivolumab and LLS30 addition served as a control cultures. Following the 24-hour incubation period, the culture medium was aspirated, and the cells were washed twice with PBS to remove any suspended PBMCs and dead 22RV1 cells. The viability of the adherent 22RV1 cells was assessed using the MTT assay.

### 2.4 Gal-1 knockdown cells establishment

PCa cells were seeded on the 6 well plates and transfected with 50 nM negative control mimic or mixed siRNA against Gal-1 or control siRNA (Qiagen)), using Lipofectamine 2000 transfection reagent (Life Technologies) according to the manufacturer’s instructions. PCa cells were infected with control or Gal-1 shRNA lentiviral particles (Santa Cruz) at an MOI of 10 for 24 h in the presence of 8 μg/ml polybrene. Immunoblotting was employed to assess Gal-1 expression following transfection with siGal-1 or shGal-1.

### 2.5 Immunoblotting analysis

Immunoblotting was done as described previously (26). Antibodies used in immunoblotting were Gal-1 (Abcam), beta-actin (Cell Signaling) and CD45 (Cell signaling). Immunoblotting bands were quantified by Image J software.

### 2.6 Quantification of secreted Gal-1 in conditioned medium from PCa cells

2 × 10^5^ 22RV1, PC3 and Myc-CaP cells were seeded per well into 6-well plates and allowed to attach for 24 hours. Following the 24-hour incubation period, cell supernatants were collected as conditioned medium. The concentration of secreted Gal-1 in the conditioned medium was measured using a Gal-1 ELISA kit (R&D Systems) following the manufacturer’s protocol.

### 2.7 Gal-1 mediated T cell apoptosis assay

Cells transfected with sicontrol or siGal-1 in culture medium were seeded onto a 6-well tissue culture plate at a density of 5×10^5^ cells per well. Following an overnight incubation, conditioned medium was obtained by centrifugation of the supernatants. To pharmacologically inhibit secreted Gal-1, LLS30 2µM was added to the conditioned medium from wild-type PCa cells. CD8+ T cells were isolated from human and mouse PBMCs using the Dynabeads CD8 Positive Isolation Kit as per the manufacturer’s instructions (Invitrogen). Then these cells were cultured in conditioned medium for 24 hours, followed by an apoptosis assay to detect Caspase-3/7 activity using a luminescent Caspase-Glo 3/7 assay kit (Promega).

### 2.8 Gal-1 binding to T cells

Fluorescein isothiocyanate (FITC) from Sigma was utilized to label recombinant Gal-1 according to the manufacturer’s instructions. This labeling occurs through the reaction of the isothiocyanate group with amino terminal and primary amines in proteins. Gal-1 at a concentration of 2 µM was treated with LLS30 at 2 µM or DMSO 0.02% for 4 hours at 4°C. T cells were collected by centrifugation and fixed in 4% paraformaldehyde. Non-specific protein binding was blocked by adding 5% BSA. T Cells were then incubated with Gal-1/LLS30 or Gal-1/DMSO. Cytospin 200 μL of the stained cell suspension onto a glass slide. The cells were mounted and photographed using a fluorescence microscope (Zeiss).

### 2.9 Co-immunoprecipitation assay

We first treated recombinant Gal-1 2 µM with LLS30 2 µM or DMSO 0.02% for 4 hours at 4°C. In addition, 2 x 10^6^ T cells were cultured for membrane protein extraction using the ProteoExtract® Native Membrane Protein Extraction Kit (Millipore). 30 μg of membrane protein was added in Gal-1/LLS30 or Gal-1/DMSO for 4 hours at 4°C. In the immunoprecipitation step, 10 µL of anti-Gal-1 (Abcam) was gently mixed with 100 µL of a Protein A/G Sepharose slurry (Abcam) and incubated for 4 hours at 4°C. Subsequently, the Sepharose slurry was added to protein mixtures with rotary agitation overnight at 4°C. Sepharose was washed with PBS, and then elution of bound proteins was performed with 150 mM glycine, pH 2.5, for 10 minutes. The eluent was then neutralized by adding 10 mL neutralization buffer (Tris, pH 8.0) and subject to immunoblotting to assess the precipitation of proteins.

### 2.10 Immunohistochemistry

Tissue sections were de-waxed using xylene twice and rehydration with 100% ethanol for 5 minutes each, followed by 95% and 80% ethanol for 5 minutes each, and then rinsed in PBS. Antigen retrieval was carried out in a 10 mM, pH 6.0 sodium citrate buffer at 95–100°C for 20 minutes. After cooling to room temperature, the sections were rinsed with PBS, and endogenous peroxidase was blocked with 1% H_2_O_2_. Non-specific binding sites were blocked with Power Block (BioGenex) for 5 minutes at room temperature. Next, the tissue sections were incubated overnight with specific antibodies against Gal-1 (Abcam), Caspase-3 (Cell Signaling), or CD8 (Cell Signaling). Following rinsing with PBS, the sections were incubated with a biotin-conjugated goat anti-rabbit IgG (BioGenex) as the second antibody. Then the sections were incubated with streptavidin-conjugated HRP (BioGenex) for 20 minutes at room temperature. HRP activity was detected using diaminobenzidine tetrahydrochloride (DAB) as the substrate (BioGenex). Nuclei were counterstained with hematoxylin (Cell Signaling). CD8-positive cells were counted in three randomly chosen areas.

### 2.11 *In vivo* xenograft tumor

LLS30 stock solutions (6X) were prepared in a mixture of 50% absolute alcohol and 50% Tween-80 to achieve a concentration of 6 mg/ml for Myc-CaP xenografts. Male FVB/N mice were obtained from the Jackson Laboratory, and 1 x 10^6^ Myc-CaP cells were subcutaneously injected into the right side of the mouse dorsal flank. The tumors were allowed to grow to approximately 100 mm^3^. Four groups of mice bearing Myc-CaP xenografts were designed, with 6 mice per group. Mice bearing Myc-CaP xenografts received intravenous administration for 2 weeks with one of the following treatments (1): vehicle control (8.7% alcohol and 8.7% Tween-80 in PBS), (2) 10 mg/kg LLS30 daily for 5 successive days, followed by a two-day break, and then for another 5 successive days, (3) 10 mg/kg anti-PD-1 given every other day for 4 shots, or (4) a combination of LLS30 and anti-PD-1 via intravenous injection. Tumor size and body weight were measured twice a week, and tumor volumes were measured using the formula (length x width^2^)/2.

### 2.12 Transcriptome sequencing and enrichment analysis

The RNA-seq data analysis involved extracting total RNA from both control and LLS30-treated 22RV1 cells using the PureLink RNA Mini Kit (Invitrogen) following the manufacturer’s instructions. RNA quality was evaluated using the Agilent 2100 Bioanalyzer system (Agilent Technologies). The mRNA sequencing library was prepared, and paired-end sequencing was conducted using the Illumina HiSeq 4000 Sequencing System. Differentially expressed genes (DEGs) were identified with a 1.5-fold change and with a significance level of P < 0.05. Volcano plots were generated using the Sigmaplot software. GO and Reactome analyses were performed on the Enrichr website (https://maayanlab.cloud/Enrichr/) using a list of gene symbols.

### 2.13 Statistical analysis

Gal-1 expression levels in tumor stroma were assessed using the following scoring system: 0 for negative, 1 for low intensity, 2 for moderate intensity, 3 for high intensity, and 4 for very high intensity. The CompuSyn software was utilized to calculate the combination index, with values below 1 indicating synergy. *In vitro* experiments were conducted in triplicate across two independent experiments, and the results are presented as the mean ± SD. The student’s t-test (two-tailed) was employed for comparing datasets between two groups with similar variance. P value < 0.05 was considered indicative of a statistically significant difference. Statistical differences, when compared with controls, are denoted as * (P < 0.05), ** (P < 0.01), or *** (P < 0.001).

## 3 Results

### 3.1 Gal-1 overexpression in the PCa TME

**Figure 1:**
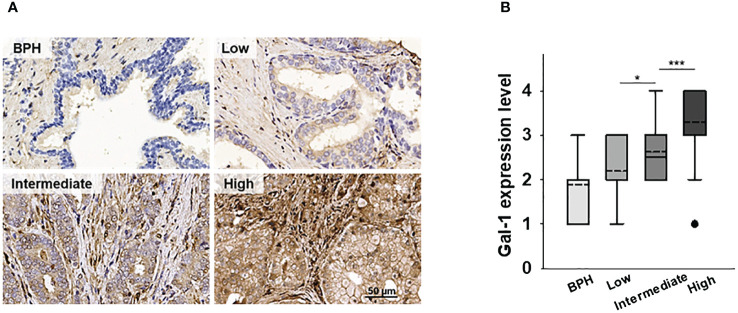
Expression of Gal-1 in the human PCa microenvironment. **(A)** The expression levels of Gal-1were detected by IHC. IHC showed that Gal-1 was localized in tumor and tumor stroma. **(B)** Tissue Gal-1 expression in 38, 32, 24 and 55 samples of BHP, low-, intermediate- and high-grade PCa tissues, respectively. Sampling distribution of Gal-1 expression was displayed by Box-Plot (dash line: mean; lines above and below the dash line, third quartile to the first quartile; lines above and below the box, maximum and minimum; dots, outlier values). *P < 0.05, ***P < 0.001; Student’s t test.

We previously demonstrated a correlation between elevated Gal-1 expression and advanced stages of PCa. However, our investigation did not encompass the assessment of Gal-1 expression in the PCa stroma. To address this gap, we reexamined our earlier TMA IHC analysis of human PCa to investigate the presence of Gal-1 in tumor stroma. The analysis results showed that, in addition to its expression in glandular epithelial cells, overexpressed Gal-1 is also observed in the stroma (**Figure 1A**). Of note, the stromal accumulation of Gal-1 exhibited a significantly progressive upregulation, transitioning from low to intermediate grades (p < 0.05 for low vs. intermediate-grade PCa) and further escalating in high-grade PCa (p < 0.001 for intermediate- vs. high-grade PCa) (**Figure 1B**). Furthermore, these findings are consistent with Castronovo’s study, wherein their dataset indicated that Gal-1 expression in cancer-associated stroma is associated with malignant tissue and tumor aggressiveness (27). These clinical observations underscore the association between increased Gal-1 expression in the stroma and the progression of PCa. This suggests that secreted Gal-1 may play a role in modulating the immune response, potentially fostering tumor progression.

### 3.2 Conditioned medium from Gal-1 expressing PCa cells induces T cell apoptosis

**Figure 2:**
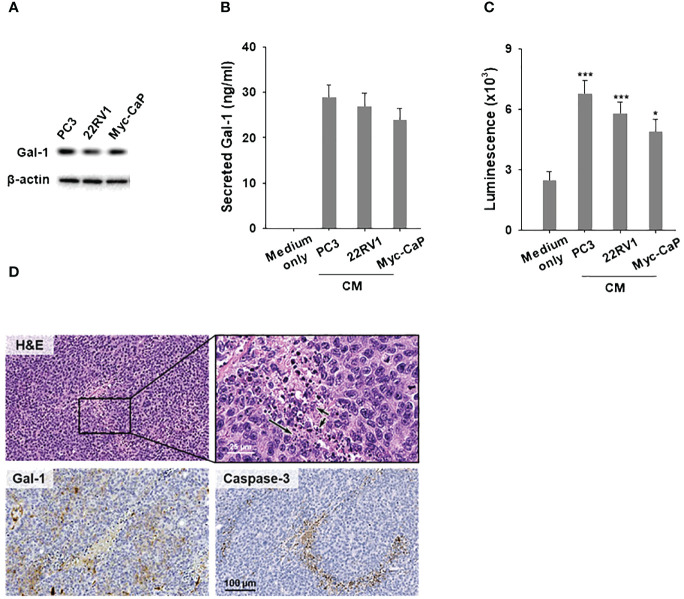
Conditioned medium from Gal-1 expressing PCa cells induces T cell apoptosis. **(A)** Immunoblotting illustrated endogenous Gal-1 expression in PCa cells. Conditioned medium (CM) from PCa cells were collected for ELISA and apoptosis assays, as depicted in **(B, C)**, respectively. **(B)** ELISA results showed the detection of secreted Gal-1 in CM from PCa cells. **(C)** Apoptosis assay for CD8^+^ T cells. CM from PCa cells induced T cell apoptosis compared to the medium-only control. **(D)** H&E and enlarged views (upper), Gal-1 staining (lower left), and Caspase-3 staining (lower right). Arrows in the image indicate the location of apoptotic cell debris with DNA fragmentation. *P < 0.05, ***P < 0.001; Student’s t test, n = 3. Data shown are mean ± s.d.

It is known that tumor-secreted Gal-1 can bind to glycosylated receptors on immune cells and trigger the apoptosis of T cells in the TME (31). However, in the context of PCa, it remains unclear whether Gal-1 can disseminate from cancer cells and induce T cell apoptosis. To investigate this, we first elucidate whether Gal-1 is secreted from cancer cells. We performed immunoblot analysis to verify the endogenous Gal-1 expression in Gal-1 expressing PCa cells including human 22RV1 (AR positive), PC3 (AR negative) and mouse Myc-CaP (AR positive) (**Figure 2A**). Following this, we cultured these PCa cells in RPMI1640 (for 22RV1 and PC3) or DMEM (for Myc-CaP), supplemented with 10% fetal bovine serum and 1% penicillin/streptomycin for 48 hours to generate conditioned medium. We then used an ELISA assay to detect the secreted Gal-1 in the conditioned medium. The ELISA results confirmed that Gal-1 was indeed secreted from PCa cells into the culture medium (**Figure 2B**). Next, conditioned medium from Gal-1 expressing cells were collected and treated with T cells to assessed for its apoptotic inducing effect on T cells. As shown in **Figure 2C**, the conditioned medium from PCa cells exerts the ability to induce apoptosis. We next determine the effects of tumor secreted Gal-1 on induced T-cell apoptosis *in vivo*. To achieve this, we subcutaneously injected the Myc-CaP cells into syngeneic FVB/N mice. Tumors were collected upon reaching a diameter of 1 cm and prepared for clinical pathology examination and IHC analysis. HE stained showed that lymphocytes have dark nuclei and a slender periphery of pale cytoplasm. Of note, we observed apoptotic cell debris with DNA fragmentation around the tumor stroma expressing Gal-1 (**Figure 2D**, basophilic nuclear remnants; arrows). Given that Caspase-3 is essential for DNA fragmentation during apoptosis, we detected caspase-3 in these tissues. IHC results confirmed the presence of caspase-3 in these tissues, supporting the apoptotic processes observed. These findings highlight the role of secreted Gal-1 in inducing T cell apoptosis in PCa.

### 3.3 Gal-1 knockdown PCa cells prevented T cell apoptosis

**Figure 3:**
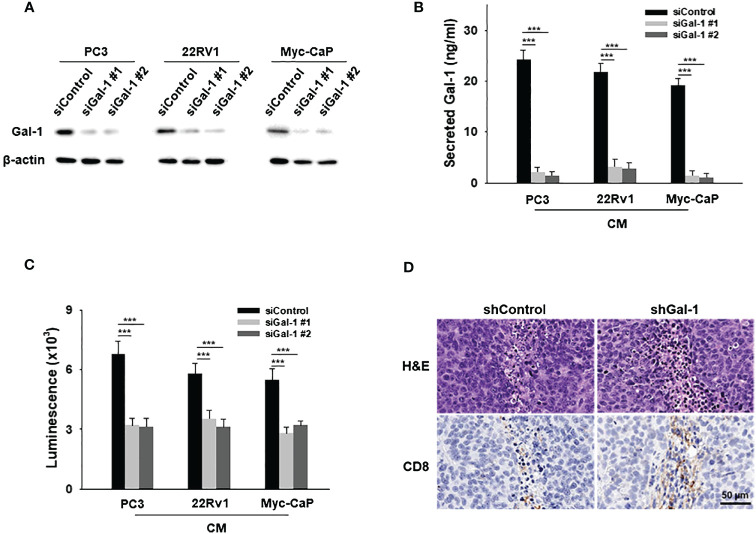
Gal-1 knockdown in PCa cells prevents T cell apoptosis. **(A)** Endogenous Gal-1 expression in PCa cells was effectively suppressed by two different siRNAs at 72 hours post-transfection. The conditioned medium (CM) from PCa were then collected for ELISA and apoptosis assays. **(B)** ELISA results illustrated the detection of secreted Gal-1 in CM from PCa sicontrol and PCa siGal-1 cells. **(C)** In the apoptosis assay for CD8^+^ T cells, CM from PCa sicontrol cells induced T cell apoptosis, but not PCa siGal-1 cells. **(D)** H&E-stained images (upper) and CD8^+^ stained images (lower). ***P < 0.001; Student’s t test, n = 3. Data shown are mean ± s.d.

To investigate the specific effects of secreted Gal-1 on T cell apoptosis, Gal-1 knockdown was conducted in Gal-1-expressing PCa cell lines. Immunoblotting confirmed the efficiency of Gal-1 knockdown (**Figure 3A**) and reduced secretion of Gal-1 as detected by ELISA (**Figure 3B**). Conditioned medium from sicontrol or siGal-1 PCa cells was then used to treat T cells. The CM from PCa Gal-1 knockdown cells showed a reduced ability to induce apoptosis compared to Gal-1 sicontrol cells (**Figure 3C**). In *in vivo* experiments, histologic analysis of the resected tumor revealed fewer apoptotic T cells around the shGal-1 tumor, indicating that Gal-1 knockdown prevents T cell apoptosis. Notably, CD8 positive cells were upregulated in the Gal-1 knockdown tumor (**Figure 3D**). These results collectively suggested that the expression of Gal-1 in the TME may induce T cell apoptosis, potentially contributing to the absence of tumor-infiltrating lymphocytes (TILs).

### 3.4 Pharmacological inhibition of secreted Gal-1 function by LLS30 suppressed T cell apoptosis

**Figure 4:**
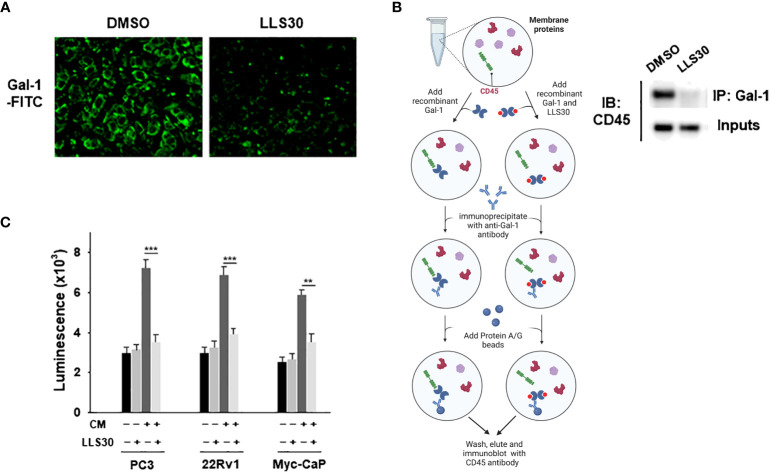
LLS30 disrupts Gal-1-carbohydrate interactions. **(A)** Cell surface glycan binding assay; 2 μM of LLS30 inhibited the adhesion of FITC labeled Gal-1 to cell surface glycan. **(B)** Left: A schematic illustrates the workflow for co-immunoprecipitation experiments analyzing the interaction between Gal-1 and CD45 after treatment with LLS30 or vehicle DMSO. Right: Co-immunoprecipitation of Gal-1 with CD45. Membrane protein extracts from T cells was treated with Gal-1/LLS30 or Gal-1/DMSO and immunoprecipitated with anti-Gal-1 antibody. Gal-1/LLS30 or Gal-1/DMSO treated T cell membrane proteins without immunoprecipitated with anti-Gal-1 antibody serve as inputs. Both inputs and the resultant immunoprecipitates were examined via immunoblotted with CD45 antibodies. **(C)** Apoptosis assay for CD8^+^ T cells. LLS30 treated CM from PCa cells suppressed induced T cell apoptosis compared to non LLS30-treated CM. **P < 0.01, ***P < 0.001; Student’s t test, n = 3. Data shown are mean ± s.d.

Extracellular Gal-1 carbohydrate recognition domain (CRD) recognized terminal galactose residues β-1,4-linked to LacNAc as presented in CD45 T cell receptors resulting in the apoptosis of effector leukocytes (17). Our previous studies showed that LLS30 binds the CRD of Gal-1 as an allosteric inhibitor leading to a decrease in binding affinity of Gal-1 to its binding partners. We further conducted a cell-based surface glycan binding assay to confirm the inhibitory effect of LLS30 on the adhesion of Gal-1 to glycosylated receptors on T cells. FITC was utilized for the labeling of Gal-1. We observed a significant decrease in fluorescence signals in the T cells in the presence of LLS30 compared to DMSO control cells (**Figure 4A**), indicating that LLS30 blocks FITC-labeled Gal-1 binding to glycosylated receptors on T cell surfaces. Given that CD45 on T cells is a primary binding partner for Gal-1 in apoptosis induction, we used Co-immunoprecipitation (co-IP) to examine whether LLS30 reduces the binding of CD45 to Gal-1. The co-IP results confirmed this reduction in CD45 binding to Gal-1 in the presence of LLS30 (**Figure 4B**). Next, we examined whether interfering Gal-1/CD45 interaction by LLS30 would reduce Gal-1-mediated apoptosis in T cells. We pretreated conditioned medium from Gal-1 wild-type PCa cells with or without LLS30 (2 µM), and co-culture with T cells to assessed the T cell apoptosis. The results showed that Gal-1 triggered T cells apoptosis can be inhibited with LLS30 at 2 µM which is not toxic for human PBMCs cells (**Figure 4C**). Taken together, these studies indicated that LLS30 binds to the Gal-1 CRD and interfere Gal-1 binding to CD45, resulting in a suppression of T cell apoptosis.

### 3.5 LLS30 potentiates antitumor activity of anti-PD1 in immunotherapy resistant PCa

**Figure 5:**
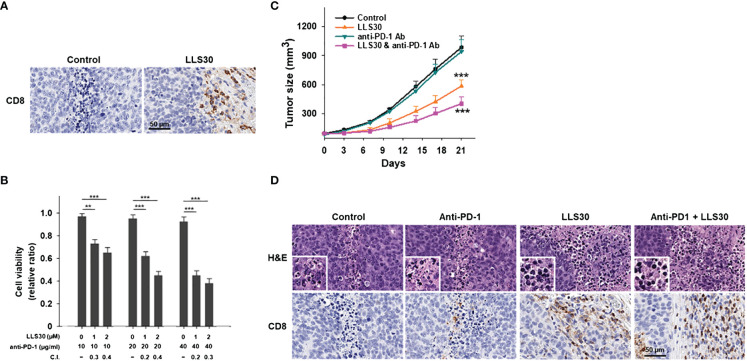
LLS30 can potentiate the anticancer effects of anti-PD1 *in vivo*. **(A)** CD8^+^ stained images after treatment with 10 µM LLS30. **(B)** Quantification of 22RV1 cells in mixed PBMCs-cancer co-cultures was conducted after 24 hours of treatment with anti-PD-1 and LLS30 at indicated concentrations. Combination index (C.I.) below 1 indicating synergy. **(C)** Tumor growth curves (n=6). Effect of LLS30, anti-PD1 and combination on growth of Myc-CaP tumor xenografts *in vivo*. **(D)** H&E and CD8^+^ stained images. Data shown are mean ± s.d. **P < 0.01, ***P< 0.001.

To assess the impact of Gal-1 inhibition by LLS30 on the TME, we subcutaneously injected the Myc-CaP cells into syngeneic FVB/N mice. We observed enhanced infiltration of CD8+ T cells in the tumors of mice treated with LLS30 (**Figure 5A**). These results suggest that LLS30 treatment prevents T cell apoptosis, resulting in the increased presence of TILs. PCa is typically considered immunologically cold tumors with minimal T cell infiltrates and limited responsiveness to checkpoint anti-PD-1 therapy. Given the increase in TILs with LLS30 treatment, we investigated whether LLS30 treatment could enhance the outcomes of anti-PD-1 therapy. We conducted *in-vitro* T cell killing assays by co-culturing 22RV1 cells with peripheral blood mononuclear cells (PBMCs). The results revealed that the combination of anti-PD-1 with LLS30 synergistically induced cell death, as evidenced by a combination index ranging from 0.2 to 0.4 (**Figure 5B**). We further assessed the combination effects on tumor growth *in vivo*. Considering the observed deficiency of T cells in Myc-CaP tumors (**Figure 5A**), we hypothesized that anti-PD1 therapy might not effectively suppress Myc-CaP tumor growth, and anticipated that inhibiting Gal-1 with LLS30 could enhance the efficacy of anti-PD1 therapy by improving T cell infiltration. *In vivo* study showed that anti-PD1 antibody alone had no effect on Myc-CaP tumor growth (**Figure 5C**). However, LLS30 at 10 mg/kg dose significantly suppressed the growth of Myc-CaP tumor, and combination of LLS30 with anti-PD-1 caused better tumor regression (**Figure 5C**). Moreover, CD-8^+^ cells were observed in LLS30-treated and combination LLS30/anti-PD1–treated tumor (**Figure 5D**).

### 3.6 LLS30 modulates cancer cell-intrinsic pathways impacting anti-tumor immunity

**Figure 6:**
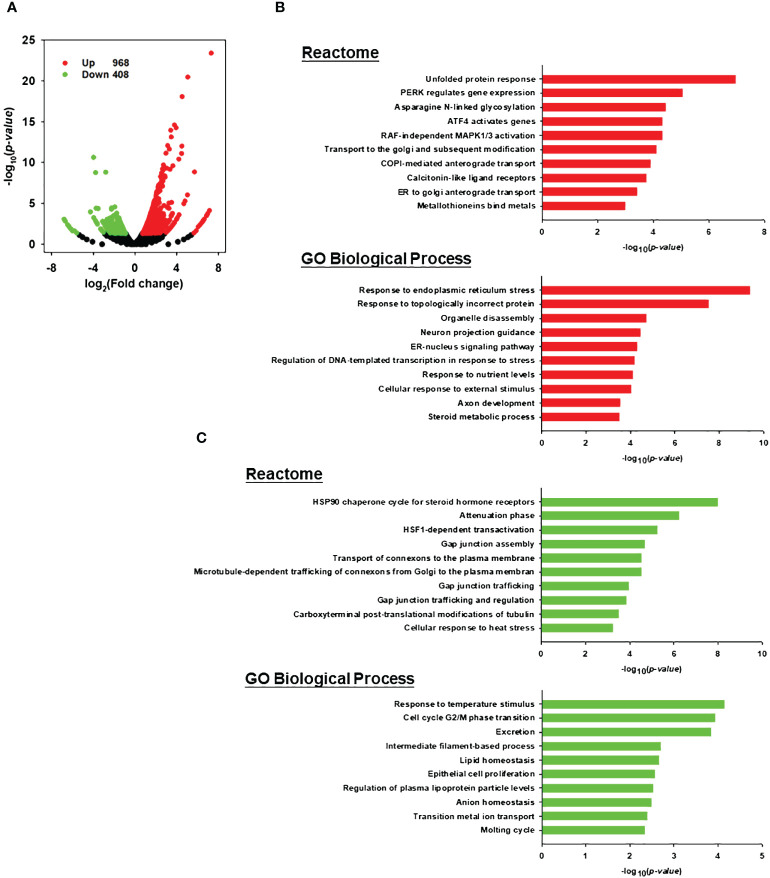
Transcriptomic analysis for LLS30 effects in 22RV1 PCa cells. **(A)** Volcano plot. The log_2_ fold change and log_10_ p-value indicated the expression level and significance for each gene. Each dot represents one gene. Black dots represent no significant differential expressed genes between control and LLS30 treatment, the green dots represent downregulated genes (FC<1.5, p<0.05) and red dots represent upregulated genes (FC>1.5, p<0.05). Reactome and GO biological process analysis of the **(B)** upregulated genes and **(C)** downregulated genes between control and LLS30 treatment. The top 10 most enriched pathways in each category were listed. Pathways significantly enriched for upregulated genes are represented by red bars, while those enriched for downregulated genes are denoted by green bars.

Increased evidence indicates that tumor-intrinsic signaling can modulate the immune response to the tumor (32). Therefore, in addition to establishing that one of the functions of LLS30 is to prevent Gal-1-mediated T cell apoptosis, we conducted a detailed exploration of the gene regulatory processes responsible for inducing cell death through the effects of LLS30. Our analysis involved examining RNA-Seq datasets to identify functional enrichments of differentially expressed genes in 22RV1 cells. RNA-Seq analysis unveiled the upregulation of 968 genes and down-regulation of 408 genes (with a 1.5-fold change and P < 0.05) following LLS30 treatment (**Figure 6A**). Functional pathway analysis performed in the differentially expressed genes revealed that LLS30 had effects on multiple important cellular pathways. In the Reactome database, the top five upregulated pathways include the unfolded protein response (UPR), PERK regulates gene expression, Asparagine N-linked glycosylation, ATF4 activates genes, and RAF-independent MAPK1/3 activation (**Figure 6B**). UPR, PERK and ATF4 are essential for induction of ER stress, suggesting LLS30 has a notable impact on induction of ER stress. Further exploration of significant pathway using the Gene Ontology (GO) biological processes database revealed that among the top five upregulated pathways, LLS30 significantly influenced ER stress (**Figure 6B**). In addition, the Reactome analysis highlighted down-regulated pathways were implicated in cellular communication and the regulation of protein folding. The GO database indicated that the down-regulated genes were associated with the maintenance of cell survival and proliferation (**Figure 6C**). Importantly, the activation of ER stress signaling has been reported to initiate a cascade of events for immunogenic apoptosis in tumor cells and subsequent recruitment and activation of T cells (33). This suggests the potential of LLS30 to induce immunogenic cell death, providing insights into its role in enhancing anti-tumor immunity and improving immunotherapy.

## 4 Discussion

Within the TME, Gal-1 significantly contributes to tumor immune evasion. In head and neck cancer, Chawla et al. discovered that 58.8% of the patient cohort exhibited moderate to marked lymphocyte infiltrates, consisting of T cells, B cells, and FoxP3-expressing T cells, while the presence of Gal-1 staining within lymphocyte areas of the tumor was significantly correlated with a poorer patient progonosis (34). In colorectal cancer, Rabinovich et al. demonstrated Gal-1 derived from the tumor promotes immunosuppression in a syngeneic colorectal cancer model by inducing CD8^+^ regulatory T cells (35). Our research findings contribute to the existing knowledge by providing evidence that Gal-1 plays a role in orchestrating an immunosuppressive microenvironment in PCa through the induction of apoptosis in effector T cells.

Tumor with poor T cell trafficking is one of the challenges for checkpoint blockade in cancer immunotherapy (36). Here we demonstrated that the inhibition of Gal-1 by LLS30 suppressed T cell apoptosis, contributing the presence of TILs and consequently enhancing the effects of anti-PD1 immunotherapy to inhibit tumor growth. Our research highlights the potential synergy between Gal-1 inhibition and an-PD-1 immunotherapy. In a recent investigation conducted by He et al., their IHC studies revealed that high Gal-1 expression was associated with a reduced number of TILs and PD-1 expression in lung, esophageal, and colorectal cancers (37). To assess the impact of combination therapy, they employed anti-PD-1 treatment in conjunction with the Gal-1 inhibitor OTX008, examining its effects on tumor growth in LLC and B16-F10 tumor models. They found that the efficacy of the combination of anti-PD-1 and Gal-1 inhibitor was significantly better than that of therapy alone. Moreover, Nambiar et al., demonstrated that expression of Gal-1 is associated with poor overall survival in head and neck cancer patients treated with immune checkpoint inhibitors (38). *In vivo* experiments also showed that Gal-1 deletion or blockade with an antibody increased intratumorally T-cell infiltration and improve the response to anti-PD1 therapy with or without radiotherapy (38). Mechanistically, Gal-1 causes immune evasion by preventing T-cell migration into tumors, by reprogramming the tumor endothelium to upregulate cell-surface PD-L1 and Gal-9 (38). Collectively, the results from these studies, indicating that Gal-1 inhibition enhances anti-PD-1 therapy, align with our findings and substantiate our notions.

Secreted Gal-1 in the TME may also function on other immune cells. Verschuere et al. demonstrated that the suppression of Gal-1 derived from glioma significantly reduced the presence of brain-infiltrating macrophages and myeloid-derived suppressor cells in an orthotopic GL261 mouse glioma model (39). Wandall et al. demonstrated that Gal-1 induces a tumor-associated macrophage-like phenotype in monocyte-derived macrophages, leading to the induced expression of PD-L1 and IDO1 in M2-like macrophages, which are key factors in immune regulation by suppressing immune responses within TME (40).

Considerable research has been conducted regarding inhibiting Gal-1 in malignancy in experimental models. Thiodigalactoside, a disaccharides, has proven effective in reducing the progression of breast cancer when administered in conjunction with vaccine immunotherapy (41–44). OTX008, a small molecule Gal-1 inhibitor, exhibits better stability compared to saccharides. Mechanistically, OTX008 inhibits ERK and causes G2/M cell cycle arrest in a panel of human cancer cell lines (28). Experimental data suggest that OTX008 alone or in combination with other standards of care may be an effective treatment for solid tumor (45, 46). In 2012, the first phase of clinical trial aimed at evaluating the effects of OTX008 for the treatment of advanced solid tumors was conducted (ClinicalTrials.gov: NCT01724320). So far, the fate of this clinical trial remains unknown because no reporting of outcome in this clinical has been released. Notably, the existing repertoire of Gal-1 inhibitors is limited in effectiveness, and none have progressed for human use. This signifies the urgency to develop a Gal-1 inhibitor that is both effective and safe for human application. To our knowledge, our group stands as the singular entity delving into the study of Gal-1 inhibition by LLS30 in PCa. We have demonstrated that LLS30 can inhibit the growth and metastasis of PCa xenografts (26). Moreover, LLS30 has the ability to potentiate the antitumor effects of docetaxel chemotherapy and anti-PD-1 immunotherapy *in vivo*. LLS30 is a promising small molecule compound that warrants further development for human clinical trials.

In conclusion, the high expression of Gal-1 in PCa TME contributes to the induction of T cell apoptosis. Importantly, by inhibiting Gal-1 function with LLS30, we successfully suppressed T cell apoptosis, resulting in increased TILs and enhanced the antitumor efficacy of anti-PD-1. Furthermore, the RNA-seq analysis results unveiled a novel mechanism of action for LLS30, highlighting a crucial link between its tumor-intrinsic oncogenic effects and anti-tumor immunity. Targeting Gal-1 with LLS30 represents a promising and novel therapeutic strategy to enhance immunotherapeutic approaches.

## Data availability statement

The data presented in the study are deposited in the BioProject database, accession number PRJNA1120033.

## Ethics statement

Human tissues were obtained from US Biomax (PR803b, 66cases) and the University of California Davis Comprehensive Cancer Center (83cases) under the protocol titled 'De-identification and Usage of Existing Pathology Specimens for Research Purposes by the UC Davis Pathology Biorepository,' with the Institutional Review Board number 293828. The animal study was approved by University of California, Davis IACUC protocols. The study was conducted in accordance with the local legislation and institutional requirements.

## Author contributions

H-CW: Conceptualization, Data curation, Formal analysis, Investigation, Methodology, Project administration, Resources, Validation, Writing – original draft, Writing – review & editing. RX: Conceptualization, Data curation, Formal analysis, Investigation, Resources, Software, Validation, Visualization, Writing – review & editing. W-HC: Data curation, Formal analysis, Investigation, Software, Writing – review & editing. S-WH: Data curation, Formal analysis, Investigation, Methodology, Software, Writing – review & editing. C-TW: Data curation, Formal analysis, Writing – review & editing. C-HC: Investigation, Methodology, Resources, Supervision, Writing – review & editing. T-CS: Conceptualization, Data curation, Formal analysis, Funding acquisition, Investigation, Methodology, Project administration, Resources, Software, Supervision, Validation, Visualization, Writing – original draft, Writing – review & editing.
